# Laparoscopic versus open liver resection for hepatocellular carcinoma in elderly patients: A systematic review and meta-analysis of propensity score-matched studies

**DOI:** 10.3389/fonc.2022.939877

**Published:** 2022-11-14

**Authors:** Shi Wang, Guanxiong Ye, Jun Wang, Shengqian Xu, Qiaoping Ye, Hailin Ye

**Affiliations:** Department of General Surgery, Lishui People’s Hospital, Lishui, China

**Keywords:** hepatocellular carcinoma (HCC), laparoscopic liver resection (LLR), open liver resection (OLR), meta-analysis, elderly

## Abstract

**Purpose:**

Laparoscopic liver resection (LLR) is a widely practiced therapeutic method and holds several advantages over open liver resection (OLR) including less postoperative pain, lower morbidity, and faster recovery. However, the effect of LLR for the treatment of hepatocellular carcinoma (HCC) in elderly patients remains controversial. Therefore, we aimed to perform the first meta-analysis of propensity score-matched (PSM) studies to compare the short- and long-term outcomes of LLR versus OLR for elderly patients with HCC.

**Methods:**

Databases including PubMed, Embase, Scopus, and Cochrane Library were systematically searched until April 2022 for eligible studies that compared LLR and OLR for the treatment of HCC in elderly patients. Short-term outcomes include postoperative complications, blood loss, surgical time, and length of hospital stay. Long-term outcomes include overall survival (OS) rate and disease-free survival (DFS) rate at 1, 3, and 5 years.

**Results:**

A total of 12 trials involving 1,861 patients (907 in the LLR group, 954 in the OLR group) were included. Compared with OLR, LLR was associated with lower postoperative complications (OR 0.49, 95% CI 0.39 to 0.62, *P* < 0.00001, *I* ^2^ = 0%), less blood loss (MD −285.69, 95% CI −481.72 to −89.65, *P* = 0.004, *I* ^2^ = 96%), and shorter hospital stay (MD −7.88, 95% CI −11.38 to −4.37, *P* < 0.0001, *I* ^2^ = 96%), whereas operation time (MD 17.33, 95% CI −6.17 to 40.83, *P* = 0.15, *I* ^2^ = 92%) was insignificantly different. Furthermore, there were no significant differences for the OS and DFS rates at 1, 3, and 5 years.

**Conclusions:**

For elderly patients with HCC, LLR offers better short-term outcomes including a lower incidence of postoperative complications and shorter hospital stays, with comparable long-term outcomes when compared with the open approach. Our results support the implementation of LLR for the treatment of HCC in elderly patients.

**Systematic review registration:**

https://inplasy.com/inplasy-2022-4-0156/, identifier INPLASY202240156.

## Introduction

Liver cancer is one of the most common cancers and a major global health challenge (1). According to GLOBOCAN 2020, liver cancer is the fourth leading cause of cancer death, causing an estimated 830,180 deaths in 2020 globally (2). Hepatocellular carcinoma (HCC) represents about 90% of primary liver cancers and constitutes a major health problem worldwide (3). Furthermore, modern advances in healthcare systems have greatly extended life expectancy (4), and the increased incidence of HCC is closely related to the aging of the population.

Surgical resection is one of the most effective treatments of choice for early HCC. Since Reich et al. reported the first laparoscopic liver resection (LLR) in 1991 (5), this minimally invasive technique has advanced continuously. Nowadays, this minimally invasive technique has gained increasing acceptance for some major well-known benefits, including a lower incidence of postoperative complications, shorter hospital stay, faster recovery, and better quality of life (6–8).

However, several factors such as the presence of comorbidities and the age of the patients may have a significant effect on the efficacy and safety of this minimally invasive technique. Age is a challenging feature given the significant heterogeneity of general conditions among individuals of the same age range and the growing number of elderly patients in good clinical condition presenting with HCC (9). Also, elderly patients are infrequently included in the range of randomized clinical trials, resulting in a lack of understanding of the benefits and risks of treatment strategies (10). Due to the factors that are mentioned above, clinicians are required to reconsider the treatment indications of this minimally invasive technique. Moreover, to surmount the existing selection and confounding biases inherent in non-randomized studies, we elected to limit to studies that performed propensity score matching (PSM), because a great number of research (11–14) have shown that PSM studies are comparable to RCTs empirically in terms of their capability of deriving unbiased estimates.

Accordingly, in order to summarize the present high-quality evidence, we performed a meta-analysis of PSM studies to compare the short- and long-term outcomes of LLR versus OLR for the treatment of HCC in elderly patients.

## Methods

We conducted our study on the basis of the updated PRISMA statement (15) (**Supplementary Material 1**), and the protocol was registered in the International Platform of Registered Systematic Review and Meta-analysis Protocols (INPLASY 202240156). We systematically searched the PubMed, Embase, Scopus, and Cochrane Library databases for PSM studies up to April 2022. The search used broad search terms containing “HCC”, “liver cancer”, “hepatoma”, “laparoscopic”, “open liver resection”, “hepatectomy”, “elderly”, and “propensity score” (the comprehensive search strategies are listed in **Supplementary Material 2**).

### Eligibility criteria

The inclusion criteria were as follows: 1) population: elderly patients (≥65 years old) with pathology‐confirmed HCC; 2) intervention: LLR; 3) comparison: OLR; 4) outcomes: short-term outcomes including postoperative complications, blood loss, surgical time, and length of hospital stay and long-term outcomes including 1-, 3-, and 5-year overall survival (OS) rates and 1-, 3-, and 5-year disease-free survival (DFS) rates; and 5) design: PSM.

### Data extraction and quality assessment

Two authors (SW and HY) independently searched relevant studies and extracted data. The characteristics of the included studies (e.g., author, years of publication, study design, population, number of patients, patient characteristics, outcomes, and covariates included in the PSM model) are recorded in **Table 1**.

**Table 1:** Characteristics of the included studies.

Study	Design	Population	Number of patients	Patient characteristics	Outcome	Covariates included in the PSM model
Monden 2022, in Japan	Single center, PSM	Patients aged ≥70 years with HCC who underwent LLR and OLR between January 2010 and June 2021	150 (LLR: 75, OLR: 75)	LLR: age 75 (70–83)^a^; male rate 71%; size of the largest tumor 24 mm (10–82)^a^; Child–Pugh A 96% OLR: age 75 (70–90)^a^; male rate 68%; size of the largest tumor 21 mm (2.7–80)^a^; Child–Pugh A 95%	Short-term outcomes: major postoperative complications, surgical time, blood loss, hospital stay, R0 resection	Age, sex, BMI, history of abdominal surgery, comorbid diseases, history of aspirin prescription, ASA classification, hepatitis status, Child–Pugh classification, tumor size, preoperative blood test, and surgical procedures
Wen 2021, in China	Single center, PSM	Patients aged over 65 with HCC who underwent liver resection between January 2015 and September 2018	142 (LLR: 71, OLR: 71)	LLR: age 68 (66, 72)^b^; male rate 76%; tumor size 5.5 cm (4.0, 7.5)^b^; liver cirrhosis 38 OLR: age: 69 (66, 72)^b^; male rate: 80%; tumor size: 6.0 cm (4.0, 8.0)^b^; liver cirrhosis 35	Short-term outcomes: postoperative complications, surgical time, blood loss, hospital stay Long-term outcomes: OS and DFS rates at 1 and 3 years	Age, sex, BMI, ASA grade, preoperative blood test, previous abdominal surgical history, comorbidities, tumor characteristics, and intraoperative records
Delvecchio 2021, in Italy, France, and Spain	Multicenter, PSM	Consecutive hepatocellular carcinoma liver resection cases in patients with ≥70 years of age	438 (LLR: 219, OLR: 219)	LLR: age 75 (70–93)^a^; male rate 72%; size of the largest tumor 35 mm (9–160)^a^; Child–Pugh A 98% OLR: age 75 (70–89)^a^; male rate 76%; size of the largest tumor 40 mm (7–150)^a^; Child–Pugh A 97%	Short-term outcomes: postoperative complications, surgical time, hospital stay Long-term outcomes: OS and DFS rates at 1, 3, and 5 years	Gender, comorbidity, ASA score, Child–Pugh score, Milan stage, number of tumors, tumor size, tumor locations, and type of hepatic resection
Nomi 2020, in Japan	Multicenter, PSM	Patients (age ≥ 75 years) who underwent liver resection for HCC between April 2010 and December 2017	310 (LLR: 155, OLR: 155)	LLR: age 78 (75–93)^a^; male rate 58%; size of the largest tumor 28 mm (2–120)^a^ OLR: age 78 (75–87)^a^; male rate 67%; size of the largest tumor 28 mm (2–150)^a^	Short-term outcomes: postoperative complications, blood loss, hospital stay, R0 resection	Sex, smoking, alcohol consumption, platelet count, underlying hepatic disease, tumor size, and type of resection
Dumronggittigule 2020, in Korea	Single center, PSM	HCC patients aged ≥70 years after hepatectomy between 2003 and 2018	82 (LLR: 41, OLR: 41)	LLR: age 73 (71, 79)^b^; male rate 68%; tumor size 3.8 cm (2.5, 6.4)^b^; Child–Pugh A 95% OLR: age 73 (71, 75)^b^; male rate 85%; tumor size 4.0 cm (2.9, 6.9)^b^; Child–Pugh A 90%	Short-term outcomes: postoperative complications, surgical time, blood loss, hospital stay, R0 resection Long-term outcomes: OS and DFS rates at 1, 3, and 5 years	Child–Turcotte–Pugh classification, tumor number, maximum size, location, extent and difficulty of liver resection
Chen 2020, in China	Single center, PSM	Patients aged 70 or over who underwent hepatectomy for HCC between January 2013 and December 2018	128 (LLR: 64, OLR: 64)	LLR: age 71 (70–77)^a^; male rate 64%; size of the largest tumor NR OLR: age 72 (70–76)^a^; male rate 59%; size of the largest tumor NR	Short-term outcomes: postoperative complications, surgical time, blood loss, hospital stay, R0 resection	Age, gender, BMI, ASA score, Charlson comorbidity index, underlying liver disease, tumor location, and type of hepatectomy
Kim 2020, in Korea	Single center, PSM	Patients older than 65 years with solitary treatment-naive HCC who underwent liver resection	182 (LLR: 91, OLR: 91)	LLR: age 70 (65–82)^a^; male rate 75%; tumor size 2.6 cm (0.9, 14.0)^a^; liver cirrhosis 44 OLR: age 69 (65–84)^a^; male rate 77%; tumor size 2.9 cm (0.3, 13.2)^a^; liver cirrhosis 47	Short-term outcomes: surgical time, blood loss, hospital stay	Tumor size, sex, protein induced by vitamin K absence or antagonist II, and cirrhosis
Badawy 2019, in Japan	Single center, PSM	Elderly patients (≥70 years) who underwent liver resection for malignant liver tumors between March 2009 and July 2016	80 (LLR: 40, OLR: 40)	LLR: age 75 (72, 79)^b^; male rate 68%; tumor size 32 mm (4–45)^a^; Child–Pugh A 98% OLR: age 76 (73, 79)^b^; male rate 58%; tumor size 24 mm (5–48)^a^; Child–Pugh A 95%	Short-term outcomes: postoperative complications, surgical time, blood loss, hospital stay Long-term outcomes: OS and DFS rates at 1, 3, and 5 years	NR
Goh 2018, in Singapore	Single center, PSM	Elderly patients (≥70 years) who underwent liver resection for HCC	64 (LLR: 32, OLR: 32)	LLR: age 73 (70–88)^a^; male rate 72%; size of the largest tumor 30 mm (14–80)^a^ OLR: age 75 (70–83)^a^; male rate 72%; size of the largest tumor 35 mm (5–90)^a^	Short-term outcomes: postoperative complications, surgical time, blood loss, hospital stay	NR
Cauchy 2016, in France	Multicenter, PSM	Elderly patients aged 65 years and older who underwent major liver resection for HCC	144 (LLR: 72, OLR: 72)	NR	Short-term outcomes: postoperative complications	Sex, age, ASA score, BMI, comorbidities, presence of severe underlying fibrosis, indication for hepatectomy tumor characteristics, type of resection, and extent of resection
Wang 2015, in China	Single center, PSM	Elderly patients (≥70 years) who underwent LLR or OLR for malignant liver carcinoma	90 (LLR: 30, OLR: 60)	LLR: age 71 (70–81)^a^; male rate 83%; size of the largest tumor 4 cm (1.5–10)^a^; Child–Pugh A 100% OLR: age 73 (71–84)^a^; male rate 75%; size of the largest tumor 5 cm (2–10)^a^; Child–Pugh A 98%	Short-term outcomes: postoperative complications, surgical time, blood loss, hospital stay	Age, sex, comorbid illness, Child–Pugh class, ASA grade, tumor size, tumor location, and extent of hepatectomy
Chan 2014, in China	Single center, PSM	Patients aged ≥70 years old who received liver resections for malignant liver tumors between January 2002 and December 2012	51 (LLR: 17, OLR: 34)	LLR: age 73 (70–94)^a^; male rate 59%; size of the largest tumor 3 cm (0.8–9.5)^a^; Child–Pugh A 100% OLR: age 74 (70–83)^a^; male rate 59%; size of the largest tumor 3 cm (1–10)^a^; Child–Pugh A 97%	Short-term outcomes: postoperative complications; surgical time, blood loss, hospital stay	Age, tumor size, and tumor location

HCC, hepatocellular carcinoma; LLR, laparoscopic liver resection; OLR, open liver resection; ASA, American Society of Anesthesiologists; OS, overall survival; DFS, disease-free survival; N, number of studies.

a Data presented as median and range.

b Data presented as median and interquartile range.

Two authors (GY and SW) independently evaluated the methodological quality of the included studies by using the Newcastle–Ottawa Scale for cohort studies. The Newcastle–Ottawa Scale contains three categories (including eight subcategories), and each study is able to acquire a maximum of 9 stars. The detailed grading standards are as follows: a score of 7 to 9 stars is graded as a high-quality study, a score of 4 to 6 stars is considered an average-quality study, whereas a score of 0 to 3 stars is classified as a low-quality study.

### Statistical synthesis and analysis

We computed the pooled odds ratio (OR) with 95% confidence interval (CI) for dichotomous outcomes and the mean difference (MD) with 95% CI for continuous outcomes. For survival data, we used the hazard ratio (HR) with 95% CI reported in the included studies. If the HR data were not reported in the original study, we imputed the HR by digitizing the Kaplan–Meier survival curves (16). The heterogeneity between studies was assessed by the Higgins inconsistency (*I* ^2^) statistics (17). Substantial heterogeneity was identified when the *I* ^2^ value >30%, and a random-effects model was employed to perform the analysis; otherwise, a fixed-effects model would be used. Funnel plots were generated to assess the possibility of publication bias, and the Egger regression test was used to measure funnel plot asymmetry (18). We considered *P <*0.05 to be statistically significant and *P <*0.10 as an indicator of trends.

Subgroup analysis stratified by types of hepatectomy [minor versus major hepatectomy, based on the Second International Consensus Conference on Laparoscopic Liver Resections (19)] and age groups (≥65, ≥70, or ≥75) was performed to investigate the potential source of heterogeneity. Finally, a sensitivity analysis was conducted to explore the effect of an individual study by the consecutive exclusion of each study at one time.

## Results

### Study identification and characteristics

**Figure 1:**
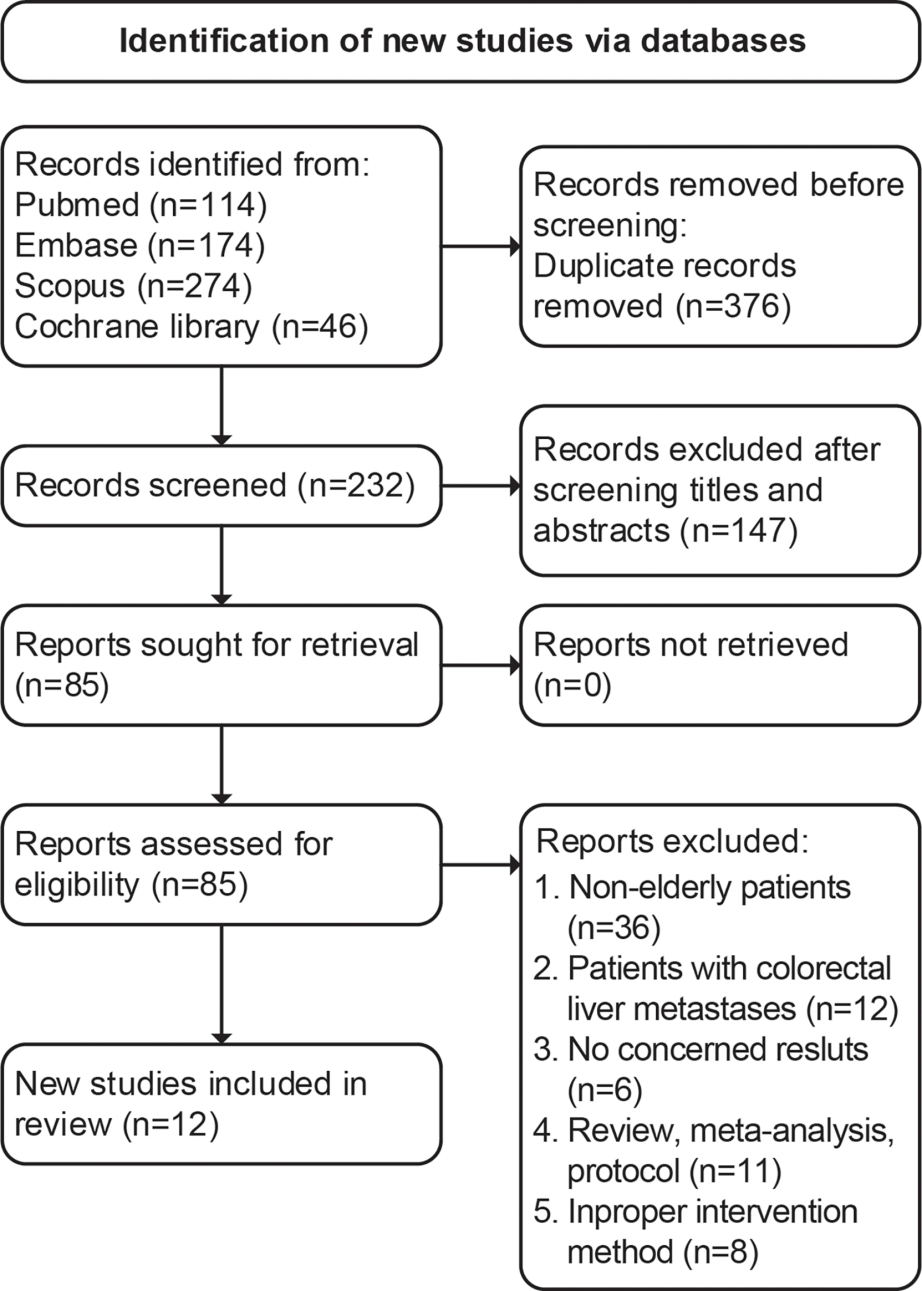
PRISMA 2020 flow diagram for the meta-analysis.

The initial search identified 608 articles (114 from PubMed, 174 from Embase, 274 from Scopus, and 46 from Cochrane Library). Among them, 376 were duplicated articles, and 147 studies were excluded by screening the abstracts. During the evaluation of the full text, 73 studies were further removed for various reasons. Eventually, a total of 12 trials (20–31) involving 1,861 patients (LLR versus OLR: 907 versus 954) were included in our study (flowchart in **Figure 1**).

**Table 1** presents the characteristics of the included studies. The number of patients in each study ranged from a minimum of 51 to 438. Among the 12 included studies, four were performed in China (22, 23, 29, 30), three in Japan (20, 27, 28), two in Korea (25, 31), one in Singapore (26), one in France (21), and one study in Italy, France, and Spain (24), respectively. Different studies define “elder patients” individually. Three studies (21, 30, 31) had an inclusion criterion of ≥65 years, eight studies (20, 22–27, 29) comprised patients aged ≥70 years, and one study (28) included patients who were 75 years old and above. The LLR and the OLR groups were comparable in terms of age, gender, characteristics of the tumor, and the American Society of Anesthesiologists score. The types of hepatectomy were diverse among each study: eight studies (20, 22, 23, 26–30) performed minor hepatectomy and four studies (21, 24, 25, 31) included minor and major hepatectomy. The postoperative complications were graded according to the Clavien–Dindo classification, and a postoperative complication of Clavien–Dindo grade ≥III was defined as a major complication (32).

In addition, the length of hospital stay, surgical time, and blood loss were expressed as median with range or interquartile range. Thus, we converted the above data into mean and standard deviation by utilizing the methodology that was developed by Wan et al. (33).

### Quality assessment

**Table 2** presents the quality assessment by the Newcastle–Ottawa Scale. All included studies had high quality with a quality score ≥7. Six studies (20, 22, 24–26, 28) did not adjust for some important confounders (such as age, sex) or the covariates included in the PSM model were not reported, and the duration of follow-up in seven studies (21–23, 26–29) was limited.

**Table 2:** Quality assessment of the included studies by the Newcastle–Ottawa Scale.

Study	Newcastle–Ottawa Scale components								Quality score
	1	2	3	4	5	6	7	8
Monden 2022	*	*	*	*	**	*		*	8
Wen 2021	*	*	*	*	**	*	*	*	9
Delvecchio 2021	*	*	*	*	*	*	*	*	8
Nomi 2020	*	*	*	*	*	*		*	7
Dumronggittigule 2020	*	*	*	*	*	*	*	*	8
Chen 2020	*	*	*	*	**	*		*	8
Kim 2020	*	*	*	*	*	*	*	*	8
Badawy 2019	*	*	*	*	*	*	*	*	8
Goh 2018	*	*	*	*	*	*		*	7
Cauchy 2016	*	*	*	*	**	*		*	8
Wang 2015	*	*	*	*	**	*		*	8
Chan 2014	*	*	*	*	*	*		*	7

1, Representativeness of the exposed cohort; 2, selection of the non-exposed cohort; 3, ascertainment of exposure; 4, demonstration that the outcome of interest was not present at the start of the study; 5, comparability of cohorts on the basis of the design or analysis; 6, assessment of outcome; 7, was follow-up long enough for outcomes to occur; 8, adequacy of follow-up of cohorts. *: get one point; **: get two points.

Funnel plots and Egger regression test for all short-term outcome measures were used to further test for potential publication bias (**Supplementary Material 3**). No significant differences were found with respect to the endpoints of postoperative complications (*P* = 0.92), blood loss (*P* = 0.4164), length of hospital stay (*P* = 0.8368), or surgical time (*P* = 0.5373). Furthermore, since the number of trials in the analysis of long-term outcomes was limited, we could not reliably assess the publication bias.

### Short-term outcomes

A total of 11 studies presented the postoperative complications (Monden et al. only reported the major postoperative complications). Overall, the incidence of postoperative complications in the LLR group was lower than that in the OLR group, 31.8% (236/741) versus 45.2% (356/788), respectively. Our meta-analysis demonstrated that LLR was associated with a lower incidence of postoperative complications (OR 0.49, 95% CI 0.39 to 0.62, *P* < 0.00001, *I* ^2^ = 0%; **Table 3**, **Supplementary Material 3**). In addition to overall postoperative complications, the incidence of pulmonary complications was significantly lower in the LLR group (OR 0.24, 95% CI 0.14 to 0.40, *P* < 0.00001, *I* ^2^ = 0%; **Table 3**, **Supplementary Material 3**). Moreover, six studies reported the rate of R0 resection, and there was no difference in the rate of R0 resection between the OLR and LLR groups (OR 1.06, 95% CI 0.30 to 3.74, *P* = 0.92, *I* ^2^ = 69%; **Table 3**, **Supplementary Material 3**).

**Table 3:** Results of this meta-analysis.

Outcome	*N*	Result (laparoscopic versus open liver resection)
**Postoperative complications**	10	OR 0.49, 95% CI 0.39 to 0.62, *P* < 0.00001, *I* ^2^ = 0%
Subgroup analysis		
Minor hepatectomy	7	OR 0.44, 95% CI 0.31 to 0.61, *P* < 0.00001, *I* ^2^ = 0%
Combined hepatectomy	3	OR 0.55, 95% CI 0.40 to 0.75, *P* = 0.0002, *I* ^2^ = 0%
		Test for subgroup difference: *I* ^2^ = 4%
Minor complications	10	OR 0.63, 95% CI 0.49 to 0.81, *P* = 0.0004, *I* ^2^ = 0%
Major complications	11	OR 0.50, 95% CI 0.36 to 0.69, *P* < 0.0001, *I* ^2^ = 0%
		Test for subgroup difference: *I* ^2^ = 23%
**Pulmonary complications**	6	OR 0.24, 95% CI 0.14 to 0.40, *P* < 0.00001, *I* ^2^ = 0%
R0 resection	6	OR 1.06, 95% CI 0.30 to 3.74, *P* = 0.92, *I* ^2^ = 69%
**Blood loss**	7	MD −285.69, 95% CI −481.72 to −89.65, *P* = 0.004, *I* ^2^ = 96%
Subgroup analysis		
Minor hepatectomy	5	MD −402.09, 95% CI −616.68 to −187.50, *P* = 0.0002, *I* ^2^ = 96%
Combined hepatectomy	2	MD 22.66, 95% CI −502.36 to 547.68, *P* = 0.93, *I* ^2^ = 91%
		Test for subgroup difference: *I* ^2^ = 54%
**Length of hospital stay**	9	MD −7.88, 95% CI −11.38 to −4.37, *P* < 0.0001, *I* ^2^ = 96%
Subgroup analysis		
Minor hepatectomy	6	MD −8.17, 95% CI −12.24 to −4.10, *P* < 0.0001, *I* ^2^ = 95%
Combined hepatectomy	3	MD −7.12, 95% CI −14.75 to 0.52, *P* = 0.07, *I* ^2^ = 97%
		Test for subgroup difference: *I* ^2^ = 0%
**Surgical time**	10	MD 17.33, 95% CI −6.17 to 40.83, *P* = 0.15, *I* ^2^ = 92%
Subgroup analysis		
Minor hepatectomy	7	MD 10.56, 95% CI −19.79 to 40.90, *P* = 0.50, *I* ^2^ = 94%
Combined hepatectomy	3	MD 39.26, 95% CI 18.97 to 59.54, *P* = 0.0001, *I* ^2^ = 35%
		Test for subgroup difference: *I* ^2^ = 58%

N, number of included studies; OR, odds ratio; CI, confidence interval; MD, mean difference; OS, overall survival; DFS, disease-free survival.

A total of seven studies reported blood loss during the operation. The meta-analysis demonstrated that LLR was associated with a significant less blood loss than OLR (MD −285.69, 95% CI −481.72 to −89.65, *P* = 0.004, *I* ^2^ = 96%; **Table 3**, **Supplementary Material 3**). Also, LLR was related to a shorter length of hospital stay (MD −7.88, 95% CI −11.38 to −4.37, *P* < 0.0001, *I* ^2^ = 96%; **Table 3**, **Supplementary Material 3**). Moreover, there was no significant difference in surgical time (MD 17.33, 95% CI −6.17 to 40.83, *P* = 0.15, *I* ^2^ = 92%; **Table 3**, **Supplementary Material 3**). However, considering the significant heterogeneity in the pooled results, the results should be interpreted with caution.

### Long-term outcomes

Four studies (20, 24, 25, 30) reported the long-term outcomes including the 1-, 3-, and 5-year OS and DFS rates, and the meta-analysis indicated that there was no significant difference in the 1-, 3-, and 5-year OS rates between the LLR and the OLR groups (1-year OS: HR 0.60, 95% CI 0.36 to 1.00, *P* = 0.05, *I* ^2^ = 7%; 3-year OS: HR 0.82, 95% CI 0.59 to 1.14, *P* = 0.24, *I* ^2^ = 0%; 5-year OS: HR 0.77, 95% CI 0.55 to 1.09, *P* = 0.15, *I* ^2^ = 20%; **Supplementary Material 3**). Similarly, the pooled results showed no significant difference in the DFS rates at 1, 3, and 5 years between the LLR and the OLR groups (1-year DFS: HR 0.65, 95% CI 0.43 to 1.00, *P* = 0.05, *I* ^2^ = 43%; 3-year DFS: HR 0.82, 95% CI 0.64to 1.04, *P* = 0.10, *I* ^2^ = 28%; 5-year DFS: HR 0.79, 95% CI 0.54 to 1.16, *P* = 0.24, *I* ^2^ = 60%; **Supplementary Material 3**).

### Subgroup and sensitivity analyses

Prespecified subgroup analyses stratified by types of hepatectomy were performed to investigate the potential discrepant treatment effect and potential sources of heterogeneity (**Table 3**, **Supplementary Material 3**). A total of eight studies (20, 22, 23, 26–30) reported patients with minor hepatectomy, and the remaining three studies (21, 24, 25) included both minor and major hepatectomy defined as the combined hepatectomy group.

The pooled ORs for postoperative complications in the two subgroups were 0.44 (95% CI 0.31 to 0.61, *P* < 0.00001, *I* ^2^ = 0%) for minor hepatectomy and 0.55 (95% CI 0.40 to 0.75, *P* = 0.0002, *I* ^2^ = 0%) for combined hepatectomy. The results indicated that LLR was associated with a lower incidence of postoperative complications for patients with minor or major hepatectomy. Moreover, for patients with minor hepatectomy, LLR was associated with less blood loss (MD −402.09, 95% CI −616.68 to −187.50, *P* = 0.0002, *I* ^2^ = 96%), shorter length of hospital stay (MD −8.17, 95% CI −12.24 to −4.10, *P* < 0.0001, *I* ^2^ = 95%), and comparable surgical time (MD 10.56, 95% CI −19.79 to 40.90, *P* = 0.50, *I* ^2^ = 94%). However, for patients with combined hepatectomy, there was no significant difference in blood loss (MD 22.66, 95% CI −502.36 to 547.68, *P* = 0.93, *I* ^2^ = 91%) and length of hospital stay (MD −7.12, 95% CI −14.75 to 0.52, *P* = 0.07, *I* ^2^ = 97%), but a longer surgical time (MD 39.26, 95% CI 18.97 to 59.54, *P* = 0.0001, *I* ^2^ = 35%) was observed. However, the significant heterogeneity and limited number of studies in this subgroup weakened the credibility of this conclusion.

Furthermore, since there were three different definitions of elderly patients (at least 60, 70, or 75 years old), we performed a subgroup analysis based on the age groups. The subgroup analysis showed that the incidence of postoperative complications was similar in three different subgroups (≥65: OR 0.36, 95% CI 0.21 to 0.61, *P* = 0.0001, *I* ^2^ = 0%; ≥70: OR 0.59, 95% CI 0.45 to 0.79, *P* = 0.0003, *I* ^2^ = 0%; ≥75: OR 0.36, 95% CI 0.21 to 0.61, *P* = 0.0002), and the subgroup of ≥70 years old showed similar results with the overall analysis. However, the subgroup of ≥65 years old showed no difference in the length of hospital stay (MD −2.26, 95% CI −4.56 to 0.03, *P* = 0.05, *I* ^2^ = 69%) but had longer surgical time (MD 40.82, 95% CI 15.29 to 66.36, *P* = 0.002, *I* ^2^ = 65%).

In addition, based on the Clavien–Dindo classification (grades I to II as minor complications, grades III to V as major complications), we divided the data of postoperative complications into minor and major complications. The results indicated that both major and minor postoperative complications were in favor of LLR (major: OR 0.50, 95% CI 0.36 to 0.69, *P* < 0.0001, *I* ^2^ = 0%; minor: OR 0.63, 95% CI 0.49 to 0.81, *P* = 0.0004, *I* ^2^ = 0%; **Table 2**, **Supplementary Material 3**).

Furthermore, the sensitivity analysis by excluding each study showed no significant difference in the short-term outcomes (**Supplementary Material 3**).

## Discussion

Considering the increase in overall life expectancy and the rising incidence of HCC, more elderly patients are considered for liver resection. Despite the advancement of laparoscopic techniques, only a few studies have focused on the potential benefits of LLR in the elderly population. In view of the scarcity of high-quality evidence, we performed this meta-analysis of PSM studies to compare the short- and long-term outcomes of LLR versus OLR for elderly patients with HCC. The results demonstrated that LLR significantly reduces postoperative complications, blood loss, and length of hospital stay, whereas the operation time was insignificantly different. Additionally, in terms of long-term survival rate, there were no significant differences between the LLR and the OLR groups. However, it should be noted that these benefits might only apply to a selected group of patients, undergoing less technically demanding minor laparoscopic hepatectomies.

Generally, the elderly are considered a vulnerable group because of the aging process, with numerous comorbidities and lower reserve capacity (34). In general, elderly patients with underlying functional status can influence the surgeons’ decision-making on surgical procedure selection. OLR for the treatment of HCC is a major abdominal surgery with high risks and difficulties, especially for elderly patients (35, 36). When choosing the clinical outcomes of our study, we compared LLR with OLR on different levels in terms of safety (postoperative complications), difficulty (operative time, blood loss), efficiency (length of hospital stays), and long-term results (OS and DFS rates). The results of our meta-analysis were broadly consistent with previous meta-analyses (35–37), indicating that LLR is a favorable approach for elderly patients that delivers improved short-term outcomes in terms of postoperative complications, blood loss, and length of hospital stay. Moreover, we further analyzed the pulmonary complications and survival rates between LLR and OLR. Our meta-analysis revealed that LLR was associated with an obviously lower incidence of pulmonary complications and no significant difference in OS or DFS rates between the LLR and the OLR groups, thereby dispelling the concerns that the laparoscopic approach may be inferior to the standard open approach in oncological efficiency.

Significantly lower rates of postoperative complications for the LLR group including a lower risk for both minor and major complications were proven in our meta-analysis. Furthermore, pulmonary complications are one of the potentially life-threatening complications after hepatectomy, especially for elderly patients. Our meta-analysis discovered a significantly lower incidence of pulmonary complications in the LLR group, and there might be several reasons for the difference. First, in open hepatectomy, the large abdominal incisions may increase the risk of wound infection and severe pain, which in turn would increase the risk of postoperative pulmonary complications. This might also be associated with delayed postoperative rehabilitation and longer hospital stay. Second, some studies have demonstrated that intraoperative fluid overload is a strong risk factor for pulmonary complications after hepatic surgery (38–40). Therefore, the lower intraoperative blood loss in the LLR group might be helpful in decreasing intraoperative fluid administration.

Another advantage of LLR is less intraoperative blood loss. The decreased blood loss in the LLR group could be attributed to the fact that the length of the incision was relatively small in laparoscopic surgery. Secondly, the hemostatic effect of the artificial pneumoperitoneum and a better view of the surgical field could also diminish blood loss (41, 42). Furthermore, the prevalence of liver cirrhosis differs among studies, but the majority is classified as Child–Pugh A, which might explain the reduced blood loss as well. Nevertheless, considering the significant heterogeneity and potential mistakes in calculating intraoperative blood loss (43), the results need to be interpreted with caution.

Concerning long-term outcomes, we observed that the laparoscopic approach had a potential long-term survival advantage, but it was not statistically significant. Moreover, it is interesting to note that the individual participant data meta-analysis of PSM studies by Syn et al. (44) demonstrated a long-term survival benefit in favor of LLR over OLR for patients with colorectal liver metastases. Although the survival benefit was not definitively confirmed in our meta-analysis, the potential clinical and biological mechanisms underlying the survival benefit associated with LLR should be attracted. First of all, many studies demonstrated that postoperative morbidity was an independent risk factor for long-term survival (45–47). The laparoscopic approach might provide a survival advantage by decreasing postoperative morbidity. Furthermore, by reducing the adverse effects of postoperative morbidity on the timing of postoperative chemotherapy, patients who had LLR have a quicker recovery and earlier resumption of chemotherapy regimens than patients with OLR (48, 49). Secondly, since laparoscopic surgery is a relatively newer surgical technique, it requires skilled surgeons with extensive experience. Thus, surgeons who routinely perform LLR may be more experienced, and the accumulated experience is associated with improved outcomes after hepatectomy for HCC (50). Moreover, the laparoscopic approach could preserve the liver parenchyma and portal pedicles or reduce the rates of dense adhesions, which may also reduce the incidence of postoperative complications and increase the feasibility of salvage surgical resection in the future (51).

However, the current study had several limitations. First and foremost, our study was limited by the retrospective and non-randomized design of the included studies. Although all included studies employed the PSM method to reduce the impact of the measured potential confounders, some unmeasured but important potential confounding factors might be overlooked. Moreover, most of the included studies had a limited sample size. Of those, nine studies were typically defined as small studies (<100 patients per arm), which may lead to a small study effect bias (52).

Secondly, there was a significant between-study heterogeneity in several outcomes, which might be derived from the differences in age ranges, liver function, number and location of lesions, general condition of the individual patient, surgeons’ experience, perioperative care protocols, pre- and postoperative chemotherapy, and other factors. Some studies included patients at wide study intervals, which may introduce biases due to advances in the mastery of surgical skills and improvements in surgical instruments (53). Noteworthy, the covariates for matching were different between the included studies, and some studies did not adjust for some important confounders such as age, sex, and liver function classification. Future research should further dissect the matching covariates to draw more accurate results.

Last but not the least, our meta-analysis only evaluated the overall and pulmonary complications. Some specific and important complications including bile leak, abscesses, and intra-abdominal infection between the two therapies could not be adequately compared, which should be further evaluated in the future.

## Conclusion

In conclusion, our meta-analysis of PSM studies suggests that LLR has improved short-term outcomes including a lower incidence of postoperative complications, less blood loss, and shorter length of hospital stay, with comparable long-term outcomes for elderly patients with HCC when compared with the open approach. However, most of the existing data are about the results of minor hepatectomy, and laparoscopic major hepatectomy in elderly patients should be carefully evaluated and preferably performed in expert centers. Furthermore, considering the limited number of included studies with small sample sizes, significant heterogeneity and potential bias were found among the included studies. Well-designed, multicenter RCTs with a large sample size are needed to further evaluate the short- and long-term outcomes of LLR versus OLR for elderly patients with HCC.

## Data availability statement

The original contributions presented in the study are included in the article/**Supplementary Material**. Further inquiries can be directed to the corresponding author.

## Author contributions

SW and HY conceived the idea, performed the analysis, and wrote the initial draft of this paper. GY, JW and SX contributed to the collection and interpretation of data. QY helped to frame the idea of the study and provided technical support. HY contributed to the revision of this paper and the final approval of the version to be published. All authors contributed to the article and approved the submitted version.

## Conflict of interest

The authors declare that the research was conducted in the absence of any commercial or financial relationships that could be construed as a potential conflict of interest.

## Publisher’s note

All claims expressed in this article are solely those of the authors and do not necessarily represent those of their affiliated organizations, or those of the publisher, the editors and the reviewers. Any product that may be evaluated in this article, or claim that may be made by its manufacturer, is not guaranteed or endorsed by the publisher.
