# Supplementary material for: Laparoscopic versus open liver resection for hepatocellular carcinoma in elderly patients: A systematic review and meta-analysis of propensity score-matched studies
Source: Front Oncol. 2022 Nov 14;12:939877. doi: 10.3389/fonc.2022.939877 (PMC9702063; doi:10.3389/fonc.2022.939877)
Supplement: Supplementary file 2 [file DataSheet_2.pdf]

Supplementary Material 2: Searching strategies

Pubmed

#1 Liver Neoplasms [MeSH] OR Carcinoma, Hepatocellular [MeSH] OR liver cancer [Title/Abstract] OR hepatoma [Title/Abstract] OR Hepatic carcinoma [Title/Abstract] OR liver carcinoma [Title/Abstract] OR hepatocellular carcinoma [Title/Abstract] OR hepatic cancer [Title/Abstract]

#2 Laparoscopic [Title/Abstract] OR Laparoscopy [MeSH] OR Laparoscopy [Title/Abstract]

#3 open [Title/Abstract]

#4 PSM [Title/Abstract] OR propensity score [Title/Abstract]

#5 Aged [MeSH] OR Elderly [Title/Abstract] OR older [Title/Abstract]

#1 AND #2 AND #3 AND #4 AND #5

Embase

#1 ‘Liver Neoplasms’:ab,ti OR ‘liver cancer’:ab,ti OR ‘Hepatic carcinoma’:ab,ti OR ‘hepatic cancer’:ab,ti OR ‘hepatocellular carcinoma’:ab,ti OR ‘liver carcinoma’:ab,ti

#2 ‘Laparoscopic’:ab,ti OR ‘Laparoscopy’:ab,ti

#3 ‘open’:ab,ti

#4 ‘PSM’:ab,ti OR ‘propensity score’:ab,ti

#5 (Aged):ab,ti OR ‘older’:ab,ti OR ‘Elderly’:ab,ti

#1 AND #2 AND #3

Scopus

#1 TITLE-ABS-KEY (Liver Neoplasms) OR TITLE-ABS-KEY (liver cancer) OR TITLE-ABS-KEY (Hepatic carcinoma) OR TITLE-ABS-KEY (hepatic cancer) OR TITLE-ABS-KEY (hepatocellular carcinoma) OR TITLE-ABS-KEY (liver carcinoma)

#2 TITLE-ABS-KEY (Laparoscopic) OR TITLE-ABS-KEY (Laparoscopy)

#3 TITLE-ABS-KEY (open)

#4 TITLE-ABS-KEY (PSM) OR TITLE-ABS-KEY (propensity score)

#5 TITLE-ABS-KEY (Aged) OR TITLE-ABS-KEY (older) OR TITLE-ABS-KEY (Elderly)

#1 AND #2 AND #3 AND #4 AND #5

Cochrane Library

#1 ‘Liver Neoplasms’:ti,ab,kw OR ‘liver cancer’: ti,ab,kw OR ‘Hepatic carcinoma’: ti,ab,kw OR ‘hepatic cancer’: ti,ab,kw OR ‘hepatocellular carcinoma’: ti,ab,kw OR ‘liver carcinoma’: ti,ab,kw

#2 Laparoscopic: ti,ab,kw OR ‘Laparoscopy’: ti,ab,kw

#3 open:ti,ab,kw

#4 (PSM): ti,ab,kw OR (propensity score): ti,ab,kw

#5 ‘Aged’: ti,ab,kw OR (older): ti,ab,kw OR (Elderly): ti,ab,kw

#1 AND #2 AND #3 AND #4 AND #5
