# Supplementary material for: Laparoscopic versus open liver resection for hepatocellular carcinoma in elderly patients: A systematic review and meta-analysis of propensity score-matched studies
Source: Front Oncol. 2022 Nov 14;12:939877. doi: 10.3389/fonc.2022.939877 (PMC9702063; doi:10.3389/fonc.2022.939877)
Supplement: Supplementary file 3 [file DataSheet_3.pdf]

**Supplementary Material 3:** Forest plots, publication bias assessment by funnel plot and Egger's test, sensitivity analyses, subgroup analyses.

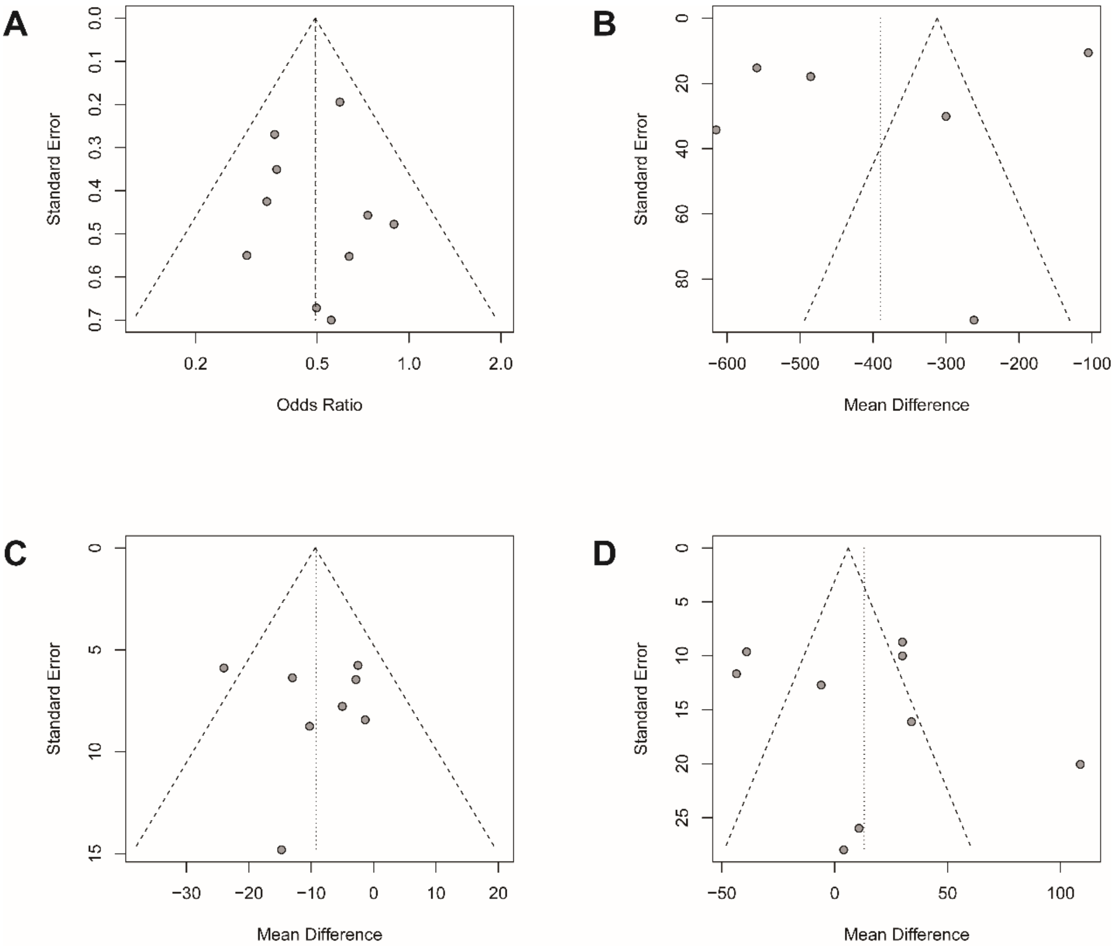


Figure 1: Funnel plot for (A) postoperative complications, Egger's test P=0.9231; (B) blood loss, Egger's test P=0.4164; (C) length of hospital stay, Egger's test P=0.8368; (D) surgical time, Egger's test P=0.5373

**A**

| Study or Subgroup | LLR Events | LLR Total | OLR Events | OLR Total | Weight | Odds Ratio M-H, Fixed, 95% CI |
|---|---|---|---|---|---|---|
| Badawy 2019 | 6 | 40 | 15 | 40 | 5.9% | 0.29 [0.10, 0.86] |
| Cauchy 2016 | 34 | 72 | 51 | 72 | 12.4% | 0.37 [0.19, 0.73] |
| Chan 2014 | 4 | 17 | 13 | 34 | 3.1% | 0.50 [0.13, 1.85] |
| Chen 2020 | 10 | 64 | 11 | 64 | 4.3% | 0.89 [0.35, 2.28] |
| Delvecchio 2021 | 82 | 219 | 110 | 219 | 31.8% | 0.59 [0.41, 0.87] |
| Dumronggittigule 2020 | 14 | 41 | 17 | 41 | 5.2% | 0.73 [0.30, 1.79] |
| Goh 2018 | 8 | 32 | 11 | 32 | 3.8% | 0.64 [0.22, 1.88] |
| Nomi 2020 | 27 | 155 | 57 | 155 | 21.7% | 0.36 [0.21, 0.61] |
| Wang 2015 | 3 | 30 | 10 | 60 | 2.8% | 0.56 [0.14, 2.19] |
| Wen 2021 | 48 | 71 | 61 | 71 | 9.1% | 0.34 [0.15, 0.79] |
| **Total (95% CI)** | | **741** | | **788** | **100.0%** | **0.49 [0.39, 0.62]** |
| Total events | 236 | | 356 | | | |

Heterogeneity: Chi² = 7.05, df = 9 (P = 0.63); I² = 0%

Test for overall effect: Z = 6.14 (P < 0.00001)

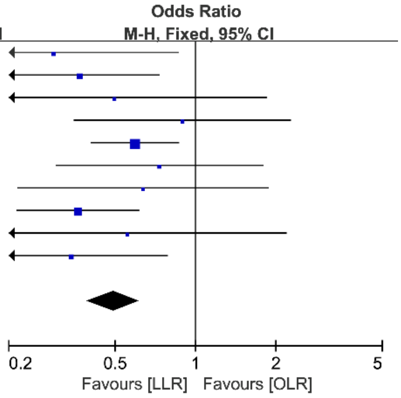


**B**

| Study or Subgroup | LLR Events | LLR Total | OLR Events | OLR Total | Weight | Odds Ratio M-H, Fixed, 95% CI |
|---|---|---|---|---|---|---|
| Badawy 2019 | 1 | 40 | 9 | 40 | 12.4% | 0.09 [0.01, 0.74] |
| Cauchy 2016 | 10 | 72 | 29 | 72 | 35.4% | 0.24 [0.11, 0.54] |
| Goh 2018 | 0 | 32 | 4 | 32 | 6.3% | 0.10 [0.01, 1.89] |
| Nomi 2020 | 5 | 155 | 18 | 155 | 24.7% | 0.25 [0.09, 0.70] |
| Wang 2015 | 1 | 30 | 3 | 60 | 2.7% | 0.66 [0.07, 6.58] |
| Wen 2021 | 5 | 71 | 14 | 71 | 18.4% | 0.31 [0.10, 0.91] |
| **Total (95% CI)** | | **400** | | **430** | **100.0%** | **0.24 [0.14, 0.40]** |
| Total events | 22 | | 77 | | | |

Heterogeneity: Chi² = 2.16, df = 5 (P = 0.83); I² = 0%

Test for overall effect: Z = 5.54 (P < 0.00001)

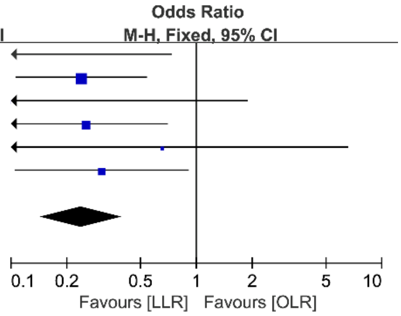


Figure 2: Forest plot for (A) overall postoperative complications; (B) pulmonary complications

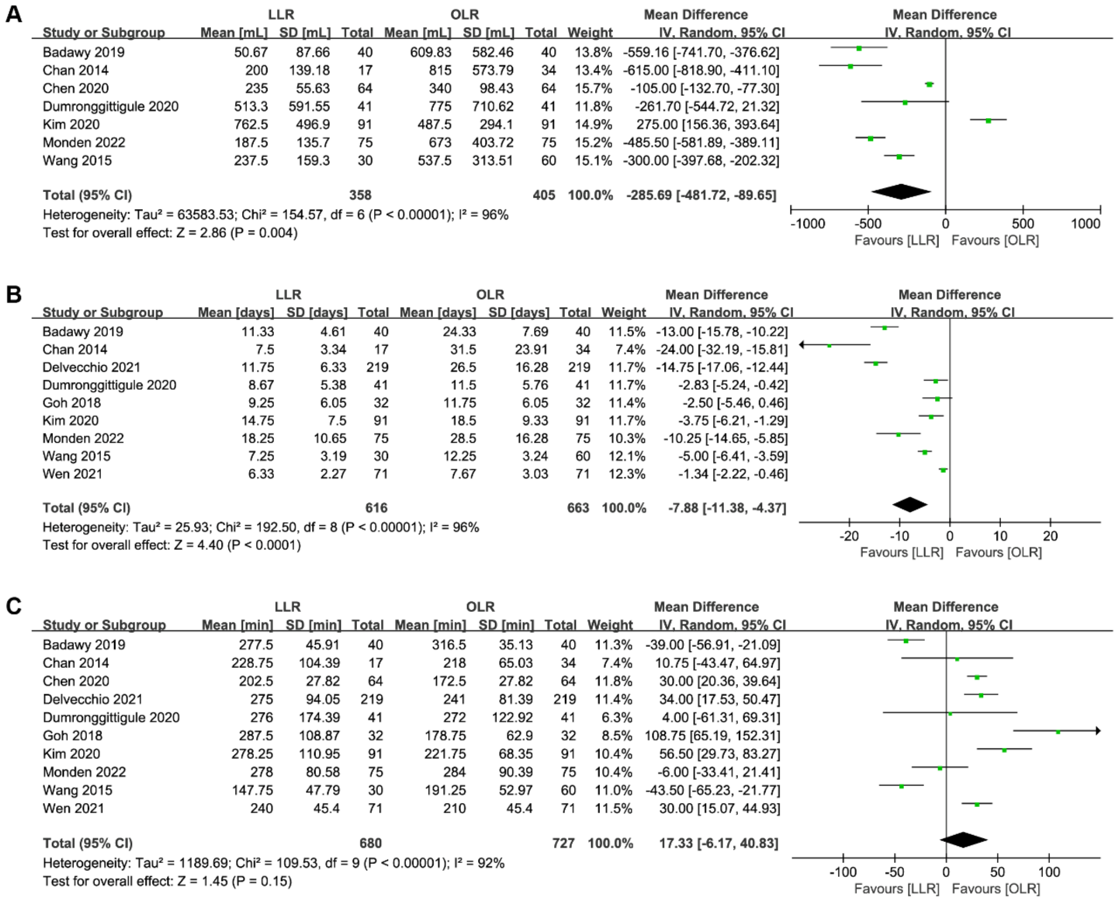


Figure 3: Forest plot for (A) blood loss; (C) length of hospital stay; (D) surgical time

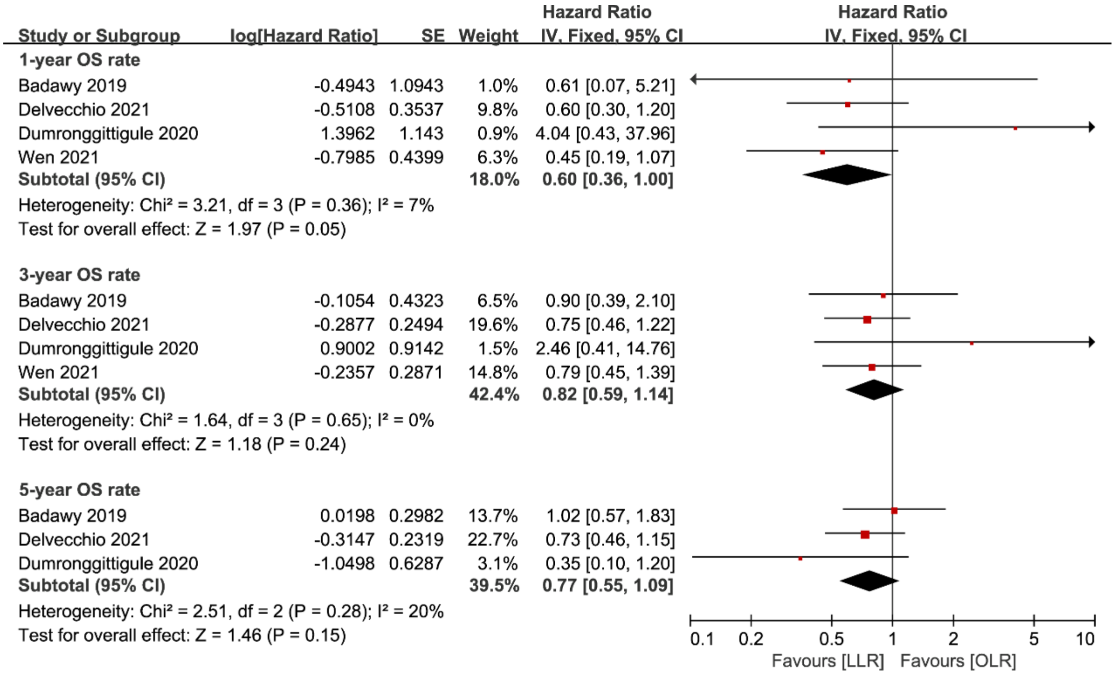


Figure 4: Forest plot for overall survival rate at 1-, 3- and 5-year

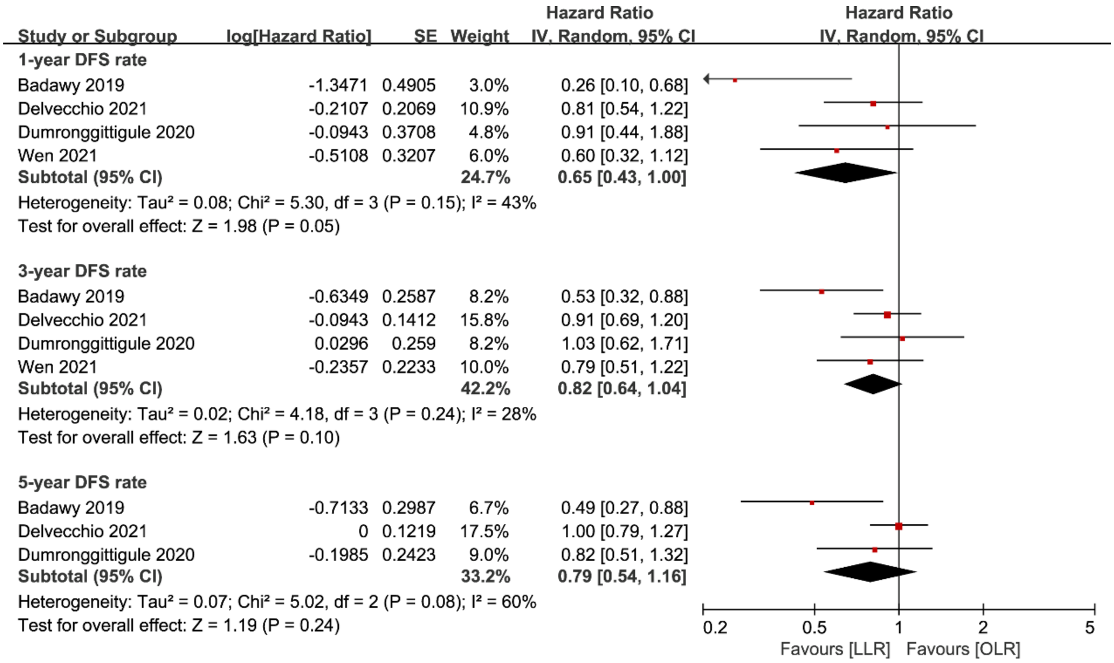


Figure 5: Forest plot for disease-free survival rate at 1-, 3- and 5-year

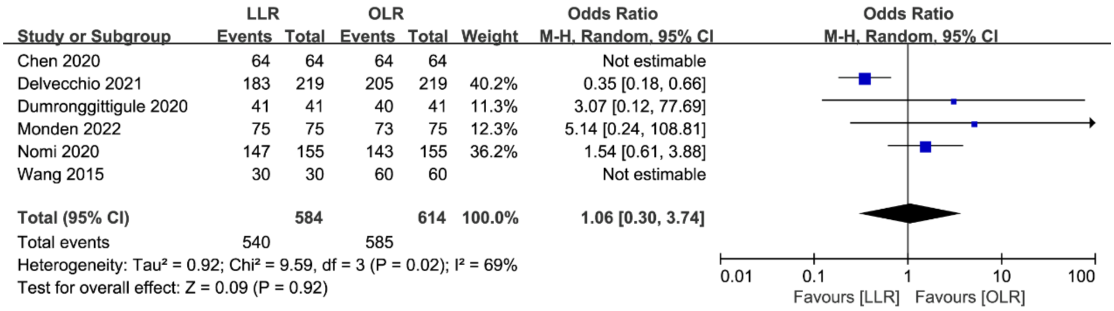


Figure 6: Forest plot for rate of R0 section

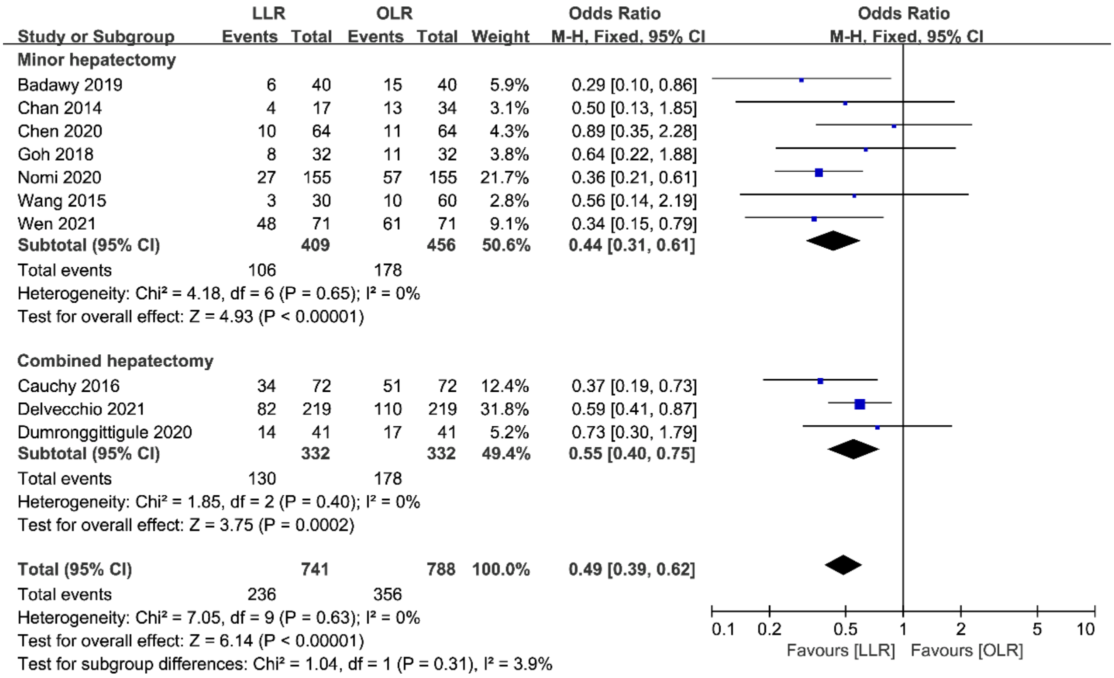


Figure 7: Subgroup analysis for postoperative complications, minor hepatectomy versus combined hepatectomy

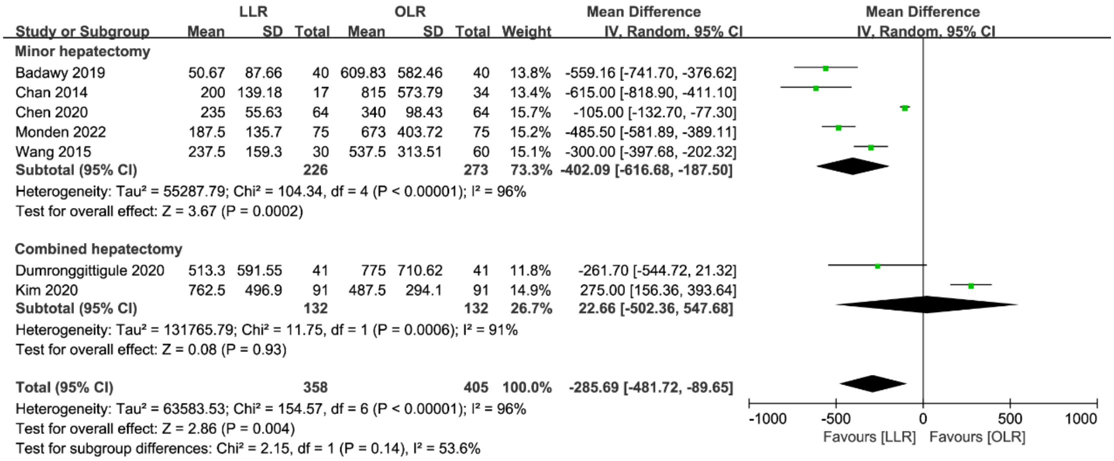


Figure 8: Subgroup analysis for blood loss, minor hepatectomy versus combined hepatectomy

| Study or Subgroup | LLR Mean | LLR SD | LLR Total | OLR Mean | OLR SD | OLR Total | Weight | Mean Difference IV, Random, 95% CI |
|---|---|---|---|---|---|---|---|---|
| **Minor hepatectomy** | | | | | | | | |
| Badawy 2019 | 11.33 | 4.61 | 40 | 24.33 | 7.69 | 40 | 11.5% | -13.00 [-15.78, -10.22] |
| Chan 2014 | 7.5 | 3.34 | 17 | 31.5 | 23.91 | 34 | 7.4% | -24.00 [-32.19, -15.81] |
| Goh 2018 | 9.25 | 6.05 | 32 | 11.75 | 6.05 | 32 | 11.4% | -2.50 [-5.46, 0.46] |
| Monden 2022 | 18.25 | 10.65 | 75 | 28.5 | 16.28 | 75 | 10.3% | -10.25 [-14.65, -5.85] |
| Wang 2015 | 7.25 | 3.19 | 30 | 12.25 | 3.24 | 60 | 12.1% | -5.00 [-6.41, -3.59] |
| Wen 2021 | 6.33 | 2.27 | 71 | 7.67 | 3.03 | 71 | 12.3% | -1.34 [-2.22, -0.46] |
| **Subtotal (95% CI)** | | | **265** | | | **312** | **64.9%** | **-8.17 [-12.24, -4.10]** |
| Heterogeneity: Tau² = 22.26; Chi² = 105.80, df = 5 (P < 0.00001); I² = 95% | | | | | | | | |
| Test for overall effect: Z = 3.94 (P < 0.0001) | | | | | | | | |
| **Combined hepatectomy** | | | | | | | | |
| Delvecchio 2021 | 11.75 | 6.33 | 219 | 26.5 | 16.28 | 219 | 11.7% | -14.75 [-17.06, -12.44] |
| Dumronggittigule 2020 | 8.67 | 5.38 | 41 | 11.5 | 5.76 | 41 | 11.7% | -2.83 [-5.24, -0.42] |
| Kim 2020 | 14.75 | 7.5 | 91 | 18.5 | 9.33 | 91 | 11.7% | -3.75 [-6.21, -1.29] |
| **Subtotal (95% CI)** | | | **351** | | | **351** | **35.1%** | **-7.12 [-14.75, 0.52]** |
| Heterogeneity: Tau² = 44.04; Chi² = 61.02, df = 2 (P < 0.00001); I² = 97% | | | | | | | | |
| Test for overall effect: Z = 1.83 (P = 0.07) | | | | | | | | |
| **Total (95% CI)** | | | **616** | | | **663** | **100.0%** | **-7.88 [-11.38, -4.37]** |
| Heterogeneity: Tau² = 25.93; Chi² = 192.50, df = 8 (P < 0.00001); I² = 96% | | | | | | | | |
| Test for overall effect: Z = 4.40 (P < 0.0001) | | | | | | | | |
| Test for subgroup differences: Chi² = 0.06, df = 1 (P = 0.81), I² = 0% | | | | | | | | |

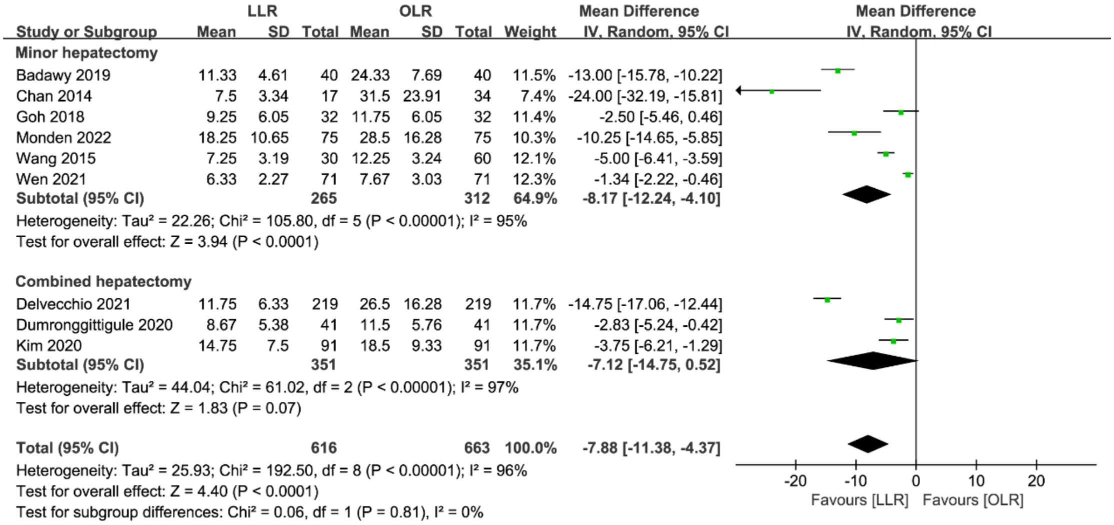


Figure 9: Subgroup analysis for length of hospital stay, minor hepatectomy versus combined hepatectomy

| Study or Subgroup | LLR Mean | LLR SD | LLR Total | OLR Mean | OLR SD | OLR Total | Weight | Mean Difference IV, Random, 95% CI |
|---|---|---|---|---|---|---|---|---|
| **Minor hepatectomy** | | | | | | | | |
| Badawy 2019 | 277.5 | 45.91 | 40 | 316.5 | 35.13 | 40 | 11.3% | -39.00 [-56.91, -21.09] |
| Chan 2014 | 228.75 | 104.39 | 17 | 218 | 65.03 | 34 | 7.4% | 10.75 [-43.47, 64.97] |
| Chen 2020 | 202.5 | 27.82 | 64 | 172.5 | 27.82 | 64 | 11.8% | 30.00 [20.36, 39.64] |
| Goh 2018 | 287.5 | 108.87 | 32 | 178.75 | 62.9 | 32 | 8.5% | 108.75 [65.19, 152.31] |
| Monden 2022 | 278 | 80.58 | 75 | 284 | 90.39 | 75 | 10.4% | -6.00 [-33.41, 21.41] |
| Wang 2015 | 147.75 | 47.79 | 30 | 191.25 | 52.97 | 60 | 11.0% | -43.50 [-65.23, -21.77] |
| Wen 2021 | 240 | 45.4 | 71 | 210 | 45.4 | 71 | 11.5% | 30.00 [15.07, 44.93] |
| **Subtotal (95% CI)** | | | **329** | | | **376** | **71.9%** | **10.56 [-19.79, 40.90]** |
| Heterogeneity: Tau² = 1462.38; Chi² = 95.68, df = 6 (P < 0.00001); I² = 94% | | | | | | | | |
| Test for overall effect: Z = 0.68 (P = 0.50) | | | | | | | | |
| **Combined hepatectomy** | | | | | | | | |
| Delvecchio 2021 | 275 | 94.05 | 219 | 241 | 81.39 | 219 | 11.4% | 34.00 [17.53, 50.47] |
| Dumronggittigule 2020 | 276 | 174.39 | 41 | 272 | 122.92 | 41 | 6.3% | 4.00 [-61.31, 69.31] |
| Kim 2020 | 278.25 | 110.95 | 91 | 221.75 | 68.35 | 91 | 10.4% | 56.50 [29.73, 83.27] |
| **Subtotal (95% CI)** | | | **351** | | | **351** | **28.1%** | **39.26 [18.97, 59.54]** |
| Heterogeneity: Tau² = 119.60; Chi² = 3.09, df = 2 (P = 0.21); I² = 35% | | | | | | | | |
| Test for overall effect: Z = 3.79 (P = 0.0001) | | | | | | | | |
| **Total (95% CI)** | | | **680** | | | **727** | **100.0%** | **17.33 [-6.17, 40.83]** |
| Heterogeneity: Tau² = 1189.69; Chi² = 109.53, df = 9 (P < 0.00001); I² = 92% | | | | | | | | |
| Test for overall effect: Z = 1.45 (P = 0.15) | | | | | | | | |
| Test for subgroup differences: Chi² = 2.37, df = 1 (P = 0.12), I² = 57.9% | | | | | | | | |

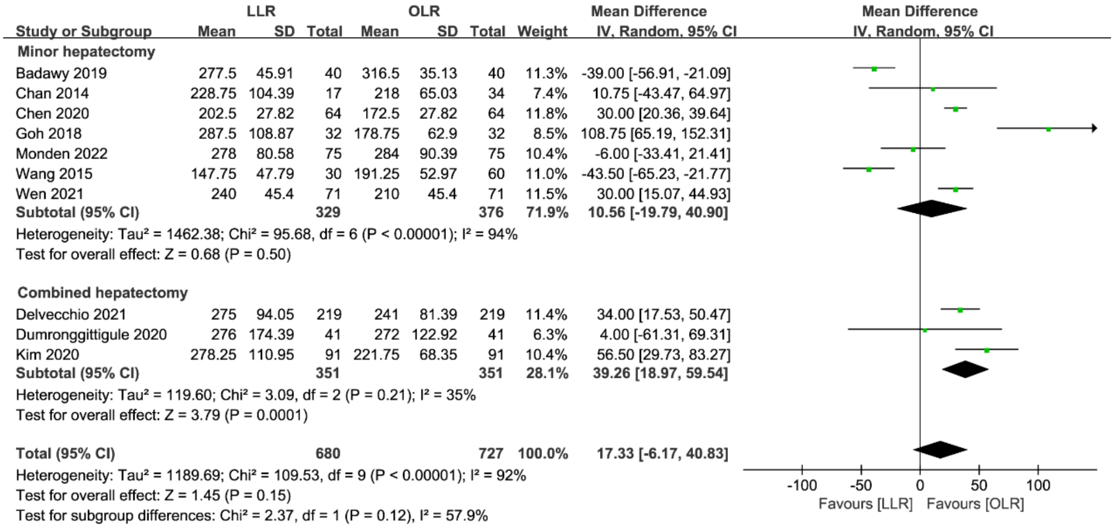


Figure 10: Subgroup analysis for surgical time, minor hepatectomy versus combined hepatectomy

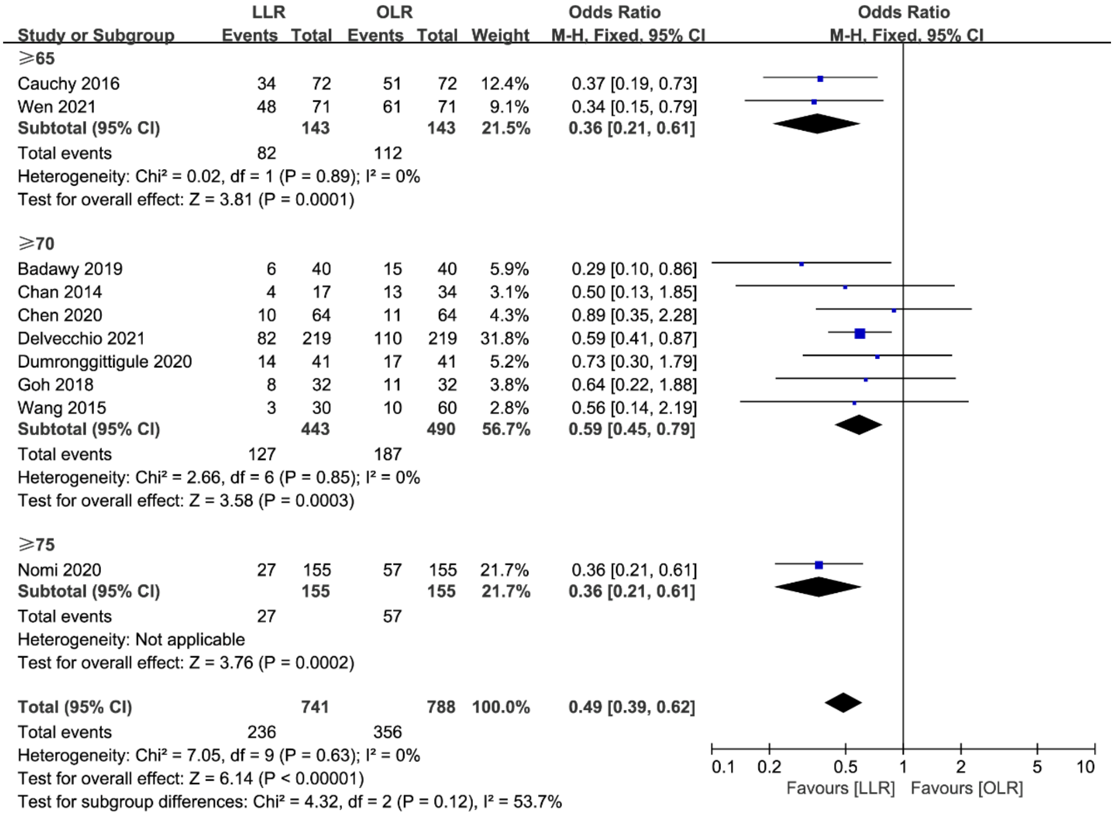


Figure 11: Subgroup analysis for postoperative complications, ≥65 versus ≥70 versus ≥75 years old

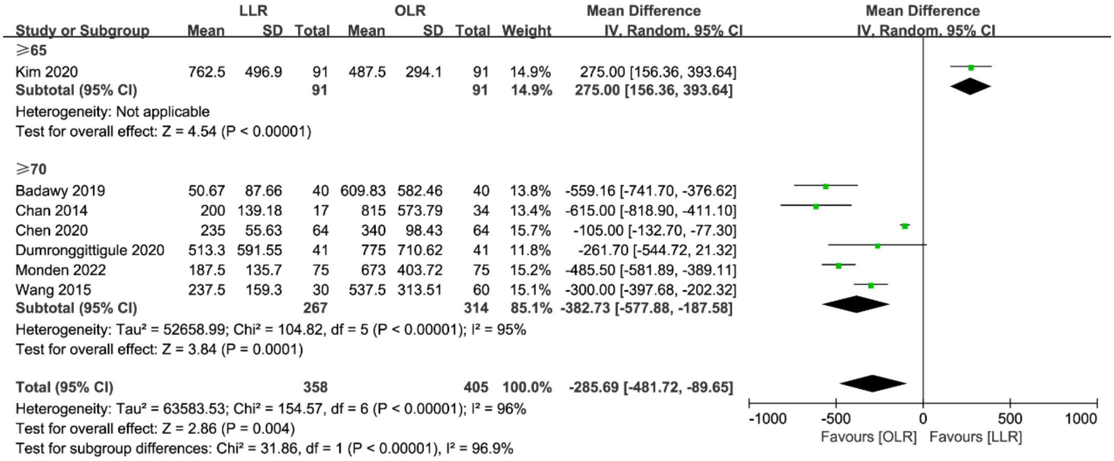


Figure 12: Subgroup analysis for blood loss, ≥65 versus ≥70 years old

| Study or Subgroup | LLR Mean | LLR SD | LLR Total | OLR Mean | OLR SD | OLR Total | Weight | Mean Difference IV, Random, 95% CI |
|---|---|---|---|---|---|---|---|---|
| ⩾65 | | | | | | | | |
| Kim 2020 | 14.75 | 7.5 | 91 | 18.5 | 9.33 | 91 | 11.7% | -3.75 [-6.21, -1.29] |
| Wen 2021 | 6.33 | 2.27 | 71 | 7.67 | 3.03 | 71 | 12.3% | -1.34 [-2.22, -0.46] |
| **Subtotal (95% CI)** | | | **162** | | | **162** | **23.9%** | **-2.26 [-4.56, 0.03]** |
| Heterogeneity: Tau² = 2.02; Chi² = 3.27, df = 1 (P = 0.07); I² = 69% | | | | | | | | |
| Test for overall effect: Z = 1.93 (P = 0.05) | | | | | | | | |
| ⩾70 | | | | | | | | |
| Badawy 2019 | 11.33 | 4.61 | 40 | 24.33 | 7.69 | 40 | 11.5% | -13.00 [-15.78, -10.22] |
| Chan 2014 | 7.5 | 3.34 | 17 | 31.5 | 23.91 | 34 | 7.4% | -24.00 [-32.19, -15.81] |
| Delvecchio 2021 | 11.75 | 6.33 | 219 | 26.5 | 16.28 | 219 | 11.7% | -14.75 [-17.06, -12.44] |
| Dumronggittigule 2020 | 8.67 | 5.38 | 41 | 11.5 | 5.76 | 41 | 11.7% | -2.83 [-5.24, -0.42] |
| Goh 2018 | 9.25 | 6.05 | 32 | 11.75 | 6.05 | 32 | 11.4% | -2.50 [-5.46, 0.46] |
| Monden 2022 | 18.25 | 10.65 | 75 | 28.5 | 16.28 | 75 | 10.3% | -10.25 [-14.65, -5.85] |
| Wang 2015 | 7.25 | 3.19 | 30 | 12.25 | 3.24 | 60 | 12.1% | -5.00 [-6.41, -3.59] |
| **Subtotal (95% CI)** | | | **454** | | | **501** | **76.1%** | **-9.63 [-13.94, -5.33]** |
| Heterogeneity: Tau² = 30.15; Chi² = 107.23, df = 6 (P < 0.00001); I² = 94% | | | | | | | | |
| Test for overall effect: Z = 4.39 (P < 0.0001) | | | | | | | | |
| **Total (95% CI)** | | | **616** | | | **663** | **100.0%** | **-7.88 [-11.38, -4.37]** |
| Heterogeneity: Tau² = 25.93; Chi² = 192.50, df = 8 (P < 0.00001); I² = 96% | | | | | | | | |
| Test for overall effect: Z = 4.40 (P < 0.0001) | | | | | | | | |
| Test for subgroup differences: Chi² = 8.78, df = 1 (P = 0.003), I² = 88.6% | | | | | | | | |

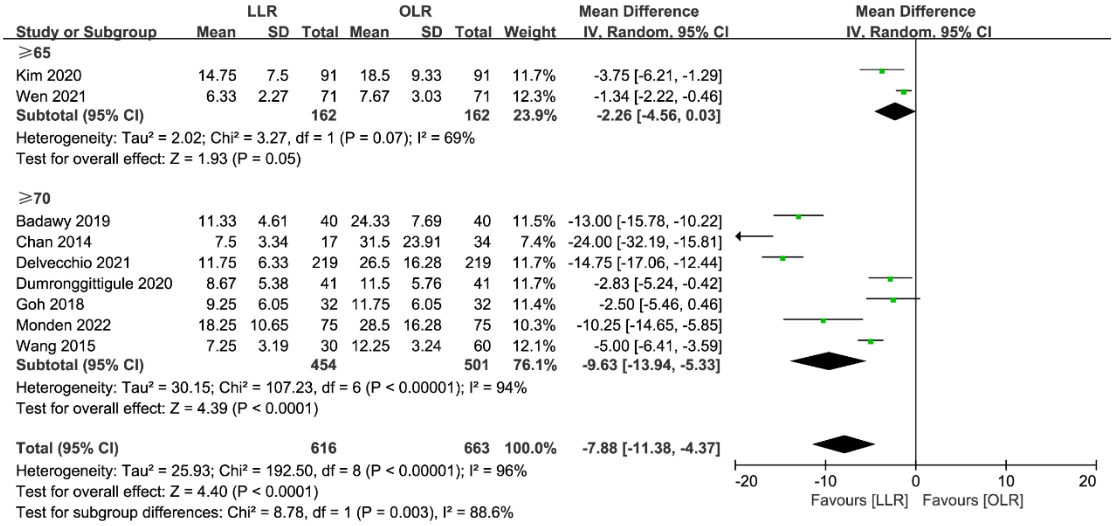


Figure 13: Subgroup analysis for length of hospital stay, ⩾65 versus ⩾70 years old

| Study or Subgroup | LLR Mean | LLR SD | LLR Total | OLR Mean | OLR SD | OLR Total | Weight | Mean Difference IV, Random, 95% CI |
|---|---|---|---|---|---|---|---|---|
| ⩾65 | | | | | | | | |
| Kim 2020 | 278.25 | 110.95 | 91 | 221.75 | 68.35 | 91 | 10.4% | 56.50 [29.73, 83.27] |
| Wen 2021 | 240 | 45.4 | 71 | 210 | 45.4 | 71 | 11.5% | 30.00 [15.07, 44.93] |
| **Subtotal (95% CI)** | | | **162** | | | **162** | **22.0%** | **40.82 [15.29, 66.36]** |
| Heterogeneity: Tau² = 228.79; Chi² = 2.87, df = 1 (P = 0.09); I² = 65% | | | | | | | | |
| Test for overall effect: Z = 3.13 (P = 0.002) | | | | | | | | |
| ⩾70 | | | | | | | | |
| Badawy 2019 | 277.5 | 45.91 | 40 | 316.5 | 35.13 | 40 | 11.3% | -39.00 [-56.91, -21.09] |
| Chan 2014 | 228.75 | 104.39 | 17 | 218 | 65.03 | 34 | 7.4% | 10.75 [-43.47, 64.97] |
| Chen 2020 | 202.5 | 27.82 | 64 | 172.5 | 27.82 | 64 | 11.8% | 30.00 [20.36, 39.64] |
| Delvecchio 2021 | 275 | 94.05 | 219 | 241 | 81.39 | 219 | 11.4% | 34.00 [17.53, 50.47] |
| Dumronggittigule 2020 | 276 | 174.39 | 41 | 272 | 122.92 | 41 | 6.3% | 4.00 [-61.31, 69.31] |
| Goh 2018 | 287.5 | 108.87 | 32 | 178.75 | 62.9 | 32 | 8.5% | 108.75 [65.19, 152.31] |
| Monden 2022 | 278 | 80.58 | 75 | 284 | 90.39 | 75 | 10.4% | -6.00 [-33.41, 21.41] |
| Wang 2015 | 147.75 | 47.79 | 30 | 191.25 | 52.97 | 60 | 11.0% | -43.50 [-65.23, -21.77] |
| **Subtotal (95% CI)** | | | **518** | | | **565** | **78.0%** | **10.60 [-18.85, 40.04]** |
| Heterogeneity: Tau² = 1509.78; Chi² = 97.02, df = 7 (P < 0.00001); I² = 93% | | | | | | | | |
| Test for overall effect: Z = 0.71 (P = 0.48) | | | | | | | | |
| **Total (95% CI)** | | | **680** | | | **727** | **100.0%** | **17.33 [-6.17, 40.83]** |
| Heterogeneity: Tau² = 1189.69; Chi² = 109.53, df = 9 (P < 0.00001); I² = 92% | | | | | | | | |
| Test for overall effect: Z = 1.45 (P = 0.15) | | | | | | | | |
| Test for subgroup differences: Chi² = 2.31, df = 1 (P = 0.13), I² = 56.7% | | | | | | | | |

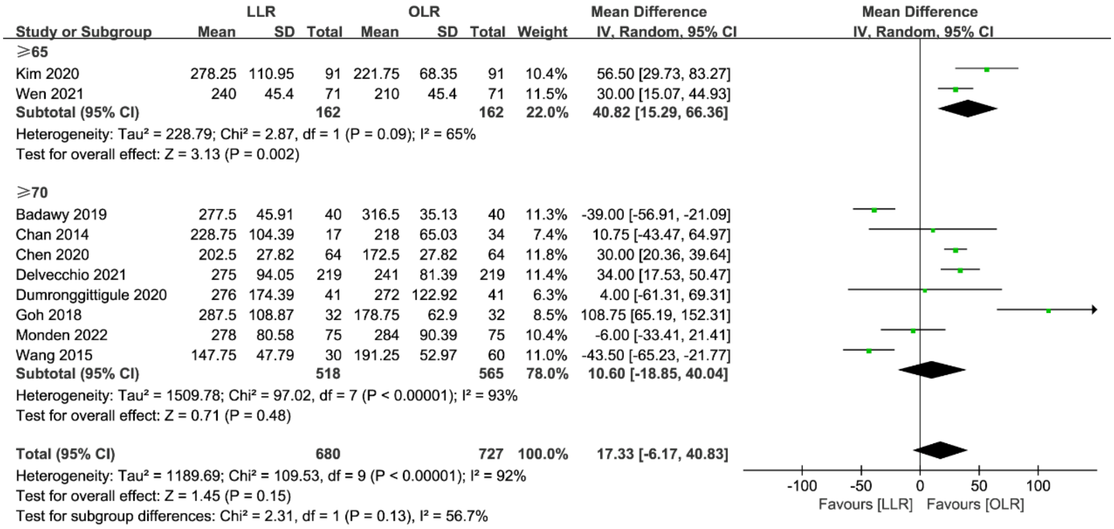


Figure 14: Subgroup analysis for surgical time, ⩾65 versus ⩾70 years old

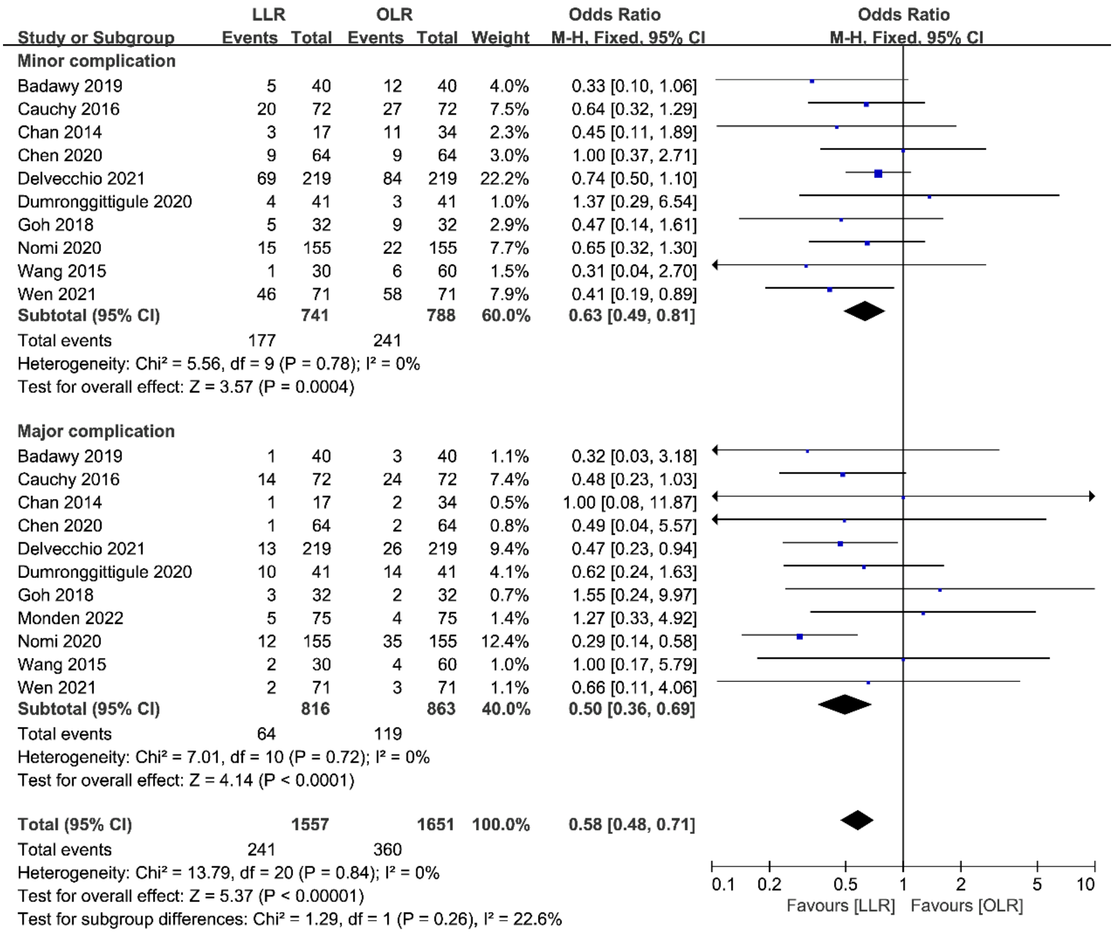


Figure 15: Subgroup analysis for postoperative complications, minor versus major complication

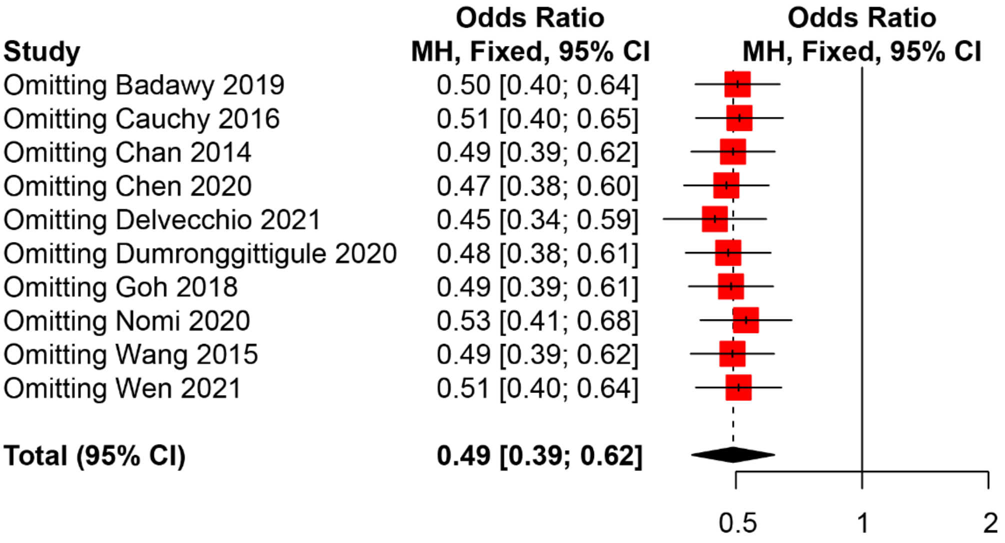


Figure 16: Sensitivity analysis for postoperative complications

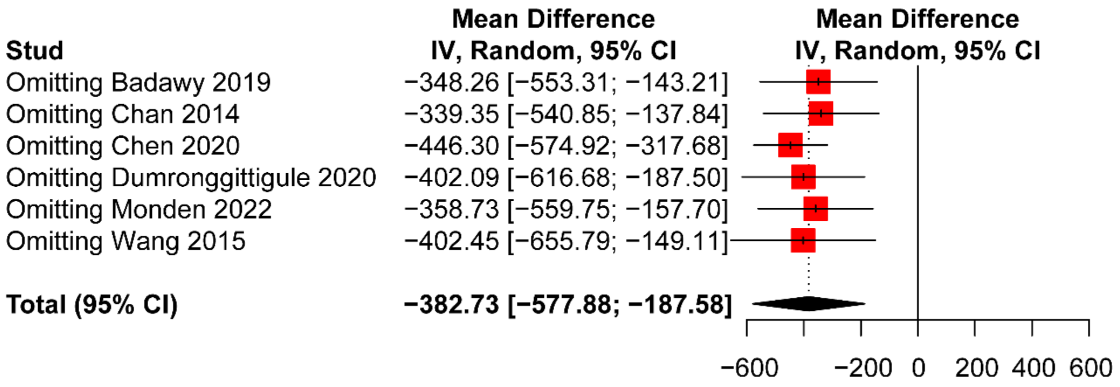


Figure 17: Sensitivity analysis for blood loss

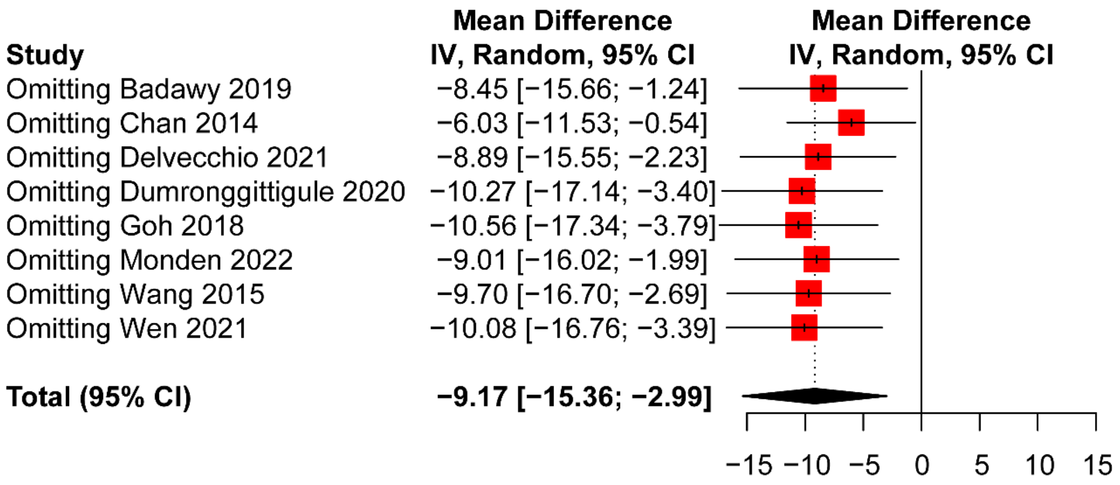


Figure 18: Sensitivity analysis for length of hospital stay

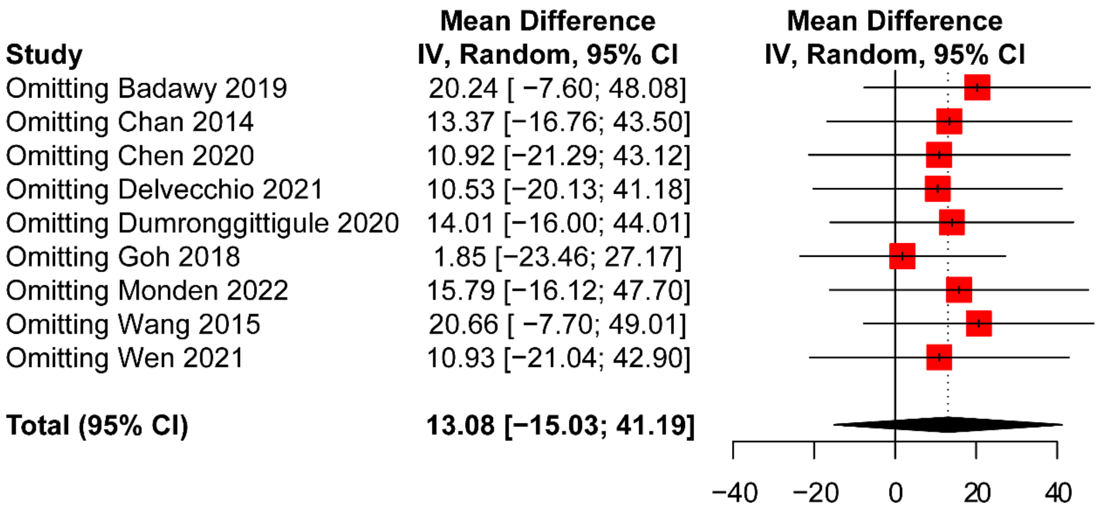


Figure 19: Sensitivity analysis for surgical time
